# Effects of lead exposure on hippocampal *metabotropic glutamate receptor subtype 3 *and *7 *in developmental rats

**DOI:** 10.1186/1477-5751-8-5

**Published:** 2009-04-20

**Authors:** Jian Xu, Huai C Yan, Bo Yang, Lu S Tong, Yu X Zou, Ying Tian

**Affiliations:** 1 Xin Hua Hospital, Shanghai Jiao Tong University School of Medicine, Shanghai Institute for Pediatric Research, Shanghai Key Laboratory of Children's Environmental Health, No. 1665 Kong Jiang Road, Shanghai 200092, PR China; 2 Shanghai Children's Medical Center, Shanghai Jiao Tong University School of Medicine, No. 1678 Dong Fang Road, Shanghai 200127, PR China; 3 Institute of Health and Biomedical Innovation, Queensland University of Technology, Kelvin Grove, Brisbane, Queensland 4059, Australia

## Abstract

**Background:**

A complete explanation of the mechanisms by which Pb^2+ ^exerts toxic effects on developmental central nervous system remains unknown. Glutamate is critical to the developing brain through various subtypes of ionotropic or metabotropic glutamate receptors (mGluRs). Ionotropic N-methyl-D-aspartate receptors have been considered as a principal target in lead-induced neurotoxicity. The relationship between *mGluR3*/*mGluR7 *and synaptic plasticity had been verified by many recent studies. The present study aimed to examine the role of *mGluR3*/*mGluR7 *in lead-induced neurotoxicity.

**Methods:**

Twenty-four adult and female rats were randomly selected and placed on control or 0.2% lead acetate during gestation and lactation. Blood lead and hippocampal lead levels of pups were analyzed at weaning to evaluate the actual lead content at the end of the exposure. Impairments of short -term memory and long-term memory of pups were assessed by tests using Morris water maze and by detection of hippocampal ultrastructural alterations on electron microscopy. The impact of lead exposure on *mGluR3 *and *mGluR7 *mRNA expression in hippocampal tissue of pups were investigated by quantitative real-time polymerase chain reaction and its potential role in lead neurotoxicity were discussed.

**Results:**

Lead levels of blood and hippocampi in the lead-exposed rats were significantly higher than those in the controls (*P *< 0.001). In tests using Morris Water Maze, the overall decrease in goal latency and swimming distance was taken to indicate that controls had shorter latencies and distance than lead-exposed rats (*P *= 0.001 and *P *< 0.001 by repeated-measures analysis of variance). On transmission electron microscopy neuronal ultrastructural alterations were observed and the results of real-time polymerase chain reaction showed that exposure to 0.2% lead acetate did not substantially change gene expression of *mGluR3 *and *mGluR7 *mRNA compared with controls.

**Conclusion:**

Exposure to lead before and after birth can damage short-term and long-term memory ability of young rats and hippocampal ultrastructure. However, the current study does not provide evidence that the expression of rat hippocampal *mGluR3 *and *mGluR7 *can be altered by systemic administration of lead during gestation and lactation, which are informative for the field of lead-induced developmental neurotoxicity noting that it seems not to be worthwhile to include *mGluR3 *and *mGluR7 *in future studies.

## Background

In spite of extensive documentation of the toxic effects of Pb^2+ ^on human health, a complete and detailed explanation of the mechanisms by which Pb^2+ ^exerts its effects on the central nervous system has not yet been found. Numerous studies have shown [1-3] that prenatal and early postnatal exposure to lead results in a long-term potentiation (LTP) decrease, cognitive deficits, and behavioral problems.

Interference with the glutamatergic neurotransmitter system has proved to be one of the key mechanisms that explains neurotoxicity of lead [4]. Glutamate is the major excitatory neurotransmitter in the mammalian brain and it mediates activity-dependent processes critical to both the developing and mature brain [4-6]. Glutamate exerts its effects through various subtypes of ionotropic or metabotropic (mGluRs) receptors [7]. Activation of the ionotropic N-methyl-D-aspartate receptors (NMDARs) plays a central role in brain development and learning and memory, which have been considered as principal consequences of lead-induced neurotoxicity [4,8-10]. However, little is known about whether mGluRs are involved in lead neurotoxicity.

mGluRs have recently been extensively studied. mGluRs are composed of eight isoforms (mGluR1~8) which are classified into groups I, II, and III. Group II (*mGluR2 and mGluR3*) and group III (*mGluR4, mGluR6, and mGluR7*) receptors are negatively coupled to adenylate cyclase by Go and possibly Gi protein [11,12]. Previous studies have shown that antagonists and agonists of mGluRs can modulate the induction, formation, and maintenance of LTP [11-15], a form of neuronal plasticity that is involved in memory and learning. The *mGluR3 *receptors are localized at high densities in brain areas associated with cognition and memory, such as the hippocampus, cortex and olfactory bulb [16-18]. Expression of *mGluR7 *is relatively high on CA3 neurons in the CA1 region [19]. The relationship between *mGluR3/mGluR7 *and synaptic plasticity had been verified by many recent studies. Pharmacological activation of *mGluR3 *revealed that *mGluR3 *may be of marked significance in the regulation of excitability in neuronal networks, as well as of synaptic plasticity [20-23]. In the study by Pöschel et al [22], activation of postsynaptic *mGluR3 *receptors were found necessary for long-term depression (LTD), presynaptic *mGluR3 *receptors functioned as modulators of both LTP and LTD [22]. On the other hand, the presynaptic axons of CA3 pyramidal neurons primarily express *mGluR7*, and *mGluR7 *modulate synaptic transmission at a variety of central synapses [24-26]. For example, Bushell et al. [24] reported that the initial decremental phase of LTP, known as short-term potentiation, was greatly attenuated in the *mGluR7 *knockout mouse (*mGluR7*-/-), which suggested a role for *mGluR7 *in short-term potentiation in the CA1 region.

We therefore undertook this study to examine the possible role of *mGluR3 *and *mGluR7 *in lead neurotoxicity. We used a whole-animal model and real-time polymerase chain reaction (PCR) to analyze the expression of *mGluR3 *and *mGluR7 *in the hippocampus of developmental rats exposed to lead during the pre- and postnatal periods. We wish to ascertain the impact of lead exposure on *mGluR3 *and *mGluR7 *expression and their potential roles in lead neurotoxicity.

## Methods

### Animal protocol and Pb^2+ ^exposure

Rats were exposed to Pb^2+ ^during development as previously described [9,27]. Briefly, twenty-four adult Sprague-Dawley rats were individually housed in plastic cages with bedding at 22 ± 2°C under a 12-hour light: dark cycle (male-female ratio 2:1, weight 200~250 g). Eight female rats were randomly selected and placed on control or 0.2% lead acetate water (Sigma-Aldrich, St. Louis, MO) from 10 days prior to mating and until postnatal day 21, namely gestational and lactational lead exposure. The lead-exposed group (4 litters) and control group (4 litters) both received the same treatment throughout the study and food and water were provided ad libitum. One day after parturition, litters were culled to 8 pups (male-female ratio 1:1) and the pups were weaned at 21 days of age. After weaning, all pups were fed deionized drinking water. All procedures complied with institutional guidelines regarding the ethical care and use of animals.

### Blood lead and hippocampal lead analysis

In each litter, four weaning pups including 2 male and 2 female rats were randomly selected to analyze the blood lead and hippocampal lead levels to evaluate the actual lead content at the end of the exposure. Blood samples (0.3–0.5 ml) were collected by cardiac puncture in tubes containing EDTA-disodium. Blood lead levels were determined via Thermo Elemental Solaar M6 Series (Thermo Elemental, Franklin, MA, USA) by Graphite Furnace Atomic Absorption Spectrometry and the quality control procedure for the assessment of lead exposure was performed. Hippocampus from both left and right sides were collected from each rat, rinsed softly with saline, sopped up water with filter paper, pooled together as one sample and weighed. After hippocampal tissues were digested by nitric acid and hydrogen peroxide, they were heated in the microwave digestion oven (CEM MARS5, USA). After that, hippocampal lead levels were measured by inductively coupled plasma mass spectrometry (ICP-MS, Agilent 7500 CE, Agilent Technologies, USA). The operations are all performed in our ICP-MS lab, which meets the Chinese Standard (GBJ173-1984) and provides air to meet class 100 (class I) conditions.

### Electron microscopy

Ultrastructural details of hippocampus were studied with electron microscopy as described [9,28]. Briefly, after 32 weaning rats (8 litters, 4 pups/litter) were sampled by cardiac puncture as above, they were immediately decapitated and collected from both sides of hippocampi and immediately cut into tissue blocks (1 mm × 1 mm × 5 mm) and were processed for electron microscopy. Ultrathin sections (50 nm) were cut with an ultramicrotome (Ultracut, Reichert-Jung) and stained with 4% uranyl acetate for 20 minutes and with 1% Pb for 10 minutes prior to examination by electron microscopy (H-500, HITACHI, Japan). The slides were read by a designated and experienced pathologist who was blinded to the dose groups.

### Morris water maze (MWM)

A test using the MWM was performed when young rats were 30 days old. In each litter, only one male and one female pups were randomly selected. The MWM was originally designed by English psychologist Morris in the 1980s [30], which consisted of a dark circular pool 150 cm in diameter and 50 cm in height. The pool was filled to a height of 35 cm with water at 22°C ± 0.5°C stained by black ink. A transparent Plexiglas^® ^escape platform (12 cm in diameter) 5 cm below the water surface and invisible to the rats was located in the center of the southwest quadrant. The room had numerous extramaze cues that remained constant throughout the experiment and no intramaze cues to ensure that the rats had to rely on the location of extramaze cues to locate the platform. The procedure included a training portion and test portion. Each training day consisted of 4 trials per animal, with a quasi-randomly selected release location from each compass point (N, E, S, W). On trial 1 of day 1, the animal was released from the appropriate starting location and once the rat located the platform it was allowed to stay on it for 10 seconds. If the rat did not find the platform within 120 seconds, it was guided to reach it and allowed to remain on it for 10 seconds and then was returned to its heated cage following completion of the task. Twenty-four hours after last training trial (postnatal day (PND) 35), 7 days later (PND 42), and 1 month later (PND72), spatial memory was repeatedly examined. On each occasion experimental procedures and surroundings were kept constant. The time required to reach the platform (escape latency), distance swimming to the platform, and the swimming speed as well as the time and distance spent in each quadrant were recorded by a video tracking system. The measures were averaged per rat within each daily session.

The MWM originally was aimed to test short-term memory (STM), namely spatial reference memory. In previous studies, the retention tests including the inhibitory avoidance task [31], hippocampal dependent discrimination task [32], and conditioned taste aversion [33], were performed to examine long-term memory (LTM) of rats which were conducted at 5 days [33], 7 days [32] or 1 month [34] after training. However, there have not any studies to assess the MWM test for evaluation of LTM. In this study, we tried to modify the classic MWM procedure and add our self-designed retention test, which might be a new and practical way to apply the MWM to evaluate LTM.

### Total RNA isolation

At 21 days of age, both sides of hippocampus of pups (8 litters, one male and one female pups/litter) were harvested and stored frozen at -80°C prepared for PCR. RNA was isolated using a Trizol kit (Invitrogen, Carlsbad, CA, USA) according to the manufacturer's instructions. Extracted RNA concentrations and purity were evaluated by measuring the A260 nm-to-A280 nm absorbance ratio with an ultraviolet spectrophotometer (Perkin Elmer, Wellesley, MA, USA). Integrity of RNA was assessed by agarose gel electrophoresis.

### Real-time reverse transcription (RT)-PCR

Highly purified oligonucleotide primers were commercially generated (SBS Genetech, China). Primer design and optimization were performed with Oligo software (National Biosciences Inc., Plymouth, MN, USA) [29]. The primers used were the following: *mGluR3 *[GenBank: M92076], sense 5'-GAC GTG GTC CTG GTG ATC CTA T-3', antisense 5'-CTA ACG GAG ATG CAC ATT G-3', 197 bp; *mGluR7 *[GenBank: D16817], sense 5'-CCA GAC AAC AAA CAC AAC CAACC-3', antisense 5'-GCG TTC CCT TCT GTG TCT TCT TC-3', 173 bp; *β-actin*, sense 5'-AGA CCT CTA TGC CAA CAC AGT GCT G-3', and antisense 5'-TCA TCG TAC TCC TGC TTG CTG A-3', 218 bp.

One-step, real-time quantitative RT-PCR was carried out with a LightCycler instrument (Roche, Mannheim, Germany) by using the LightCycler SYBR Green I RNA Master Kit (Roche, Mannheim, Germany). All reactions were conducted in duplicate. Negative control was performed with sterile purified deionized water. Each cycle of PCR included denaturation at 95°C for 5 seconds, primers annealing at 62°C for 5 seconds, and a final extension at 72°C for 12 seconds. The fluorescence of each sample was measured at 5°C below the melting temperatures (Tms) to eliminate background fluorescence due to primer-dimer [35]. Results were analyzed with LightCycler Software version 3.5 by using the second derivative maximum method to set the CT. E was calculated using the equation E = 10^(-1/slope) ^[36-38]. Agarose gel electrophoresis analyses were also performed to verify whether the amplified product corresponded to the size predicted for gene-specific product.

Relative quantification was carried out with the Relative Expression Software Tool (REST, Roche, Mannheim, Germany). Because the expression level of the *β-actin *gene was constant regardless of lead exposure [39], relative qualification was presented by means of normalization with the *β-actin *gene. Relative and normalized expression ratios (R) were calculated on the basis of the median of the performed duplicates and computed according to the following equation: R = E_target_exp(ΔC_Ttarget_)/E_ref_exp(ΔC_Tref_) [29,36,37].

### Statistical analysis

Wilcoxon test was used in the analyses [40]. The variations in *mGluR3 *and *mGluR7 *expression were compared using coefficients of variability and the Wilcoxon two group test. Blood lead levels and hippocampal lead levels were analyzed with one-way analysis of variance (ANOVA). In the MWM task, distance traveled (cm) and escape latency were the principal measures to evaluate the performance of the rats during acquisition training. The baselines of pretraining latency and swimming distance of two groups were analyzed with one-way ANOVA. Because the experimental design involves both a between-subjects factor (lead dose condition) and a within-subjects factor (days), repeated measures ANOVA was performed. Data are presented as mean ± SD and the level of significance is *P *< 0.05 (two tailed). All statistical evaluations were performed using standard statistical software (SAS Institute Inc., Cary, NC, USA).

## Results

### Blood lead and hippocampal lead analysis

Lead concentrations of blood and hippocampus were 3.0 ± 0.2 μg/dL and 51.9 ± 6.5 μg/kg, respectively, in 16 control rats and 56.8 ± 4.4 μg/dL and 432.9 ± 15.1 μg/kg, respectively, in 16 lead-exposed rats. Lead levels of blood and hippocampi in the rats exposed to lead were significantly higher than those in the controls (*n *= 16, *P *< 0.001).

### Neuronal ultrastructural alterations

**Figure 1:**
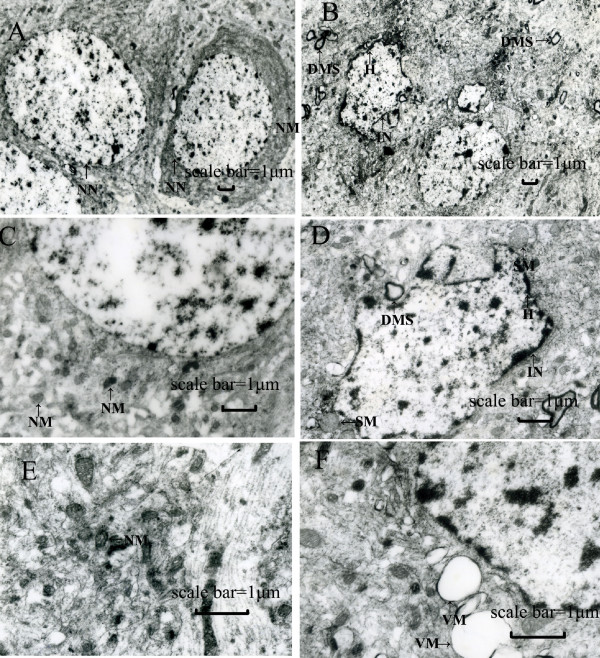
**Representative electron micrographs of coronal sections of the rat hippocampus are shown**. (**A, C**, and **E**) control hippocampus. (**B, D **and **F**) lead-exposed hippocampus in rats at weaning that were treated with 0.2% lead acetate during the gestational and lactational periods as described. Abnormal appearance of neurons including irregular shaped nucleus, swollen mitochondria, often vacuolated with disrupted cristae, a large quantity of heterochromatin collected inside the nucleus, demyelination or shrinkage, and denaturation of the myelin sheath were observed. These findings suggest that hippocampal ultrastructures were injured by lead exposure during the early stage of life. Scale bar = 1 μm. NN: normal nucleus; IN: irregular nucleus; SM: swollen mitochondria; VM: vacuolated mitochondria; NM: normal mitochondria; H: heterochromatin; DMS: denaturation of myelin sheath.

On transmission electron microscopy neuronal ultrastructural alterations, such as damage of mitochondria, microfilaments, and microtubules, were observed. Vacuole formation from swollen and distorted mitochondria, chromatin condensation, nucleolus collapse or fragmentation and myelin sheath degeneration were found in lead-exposed hippocampal neurons compared with controls (Figure 1).

### Spatial learning and memory abilities evaluated by MWM

**Figure 2:**
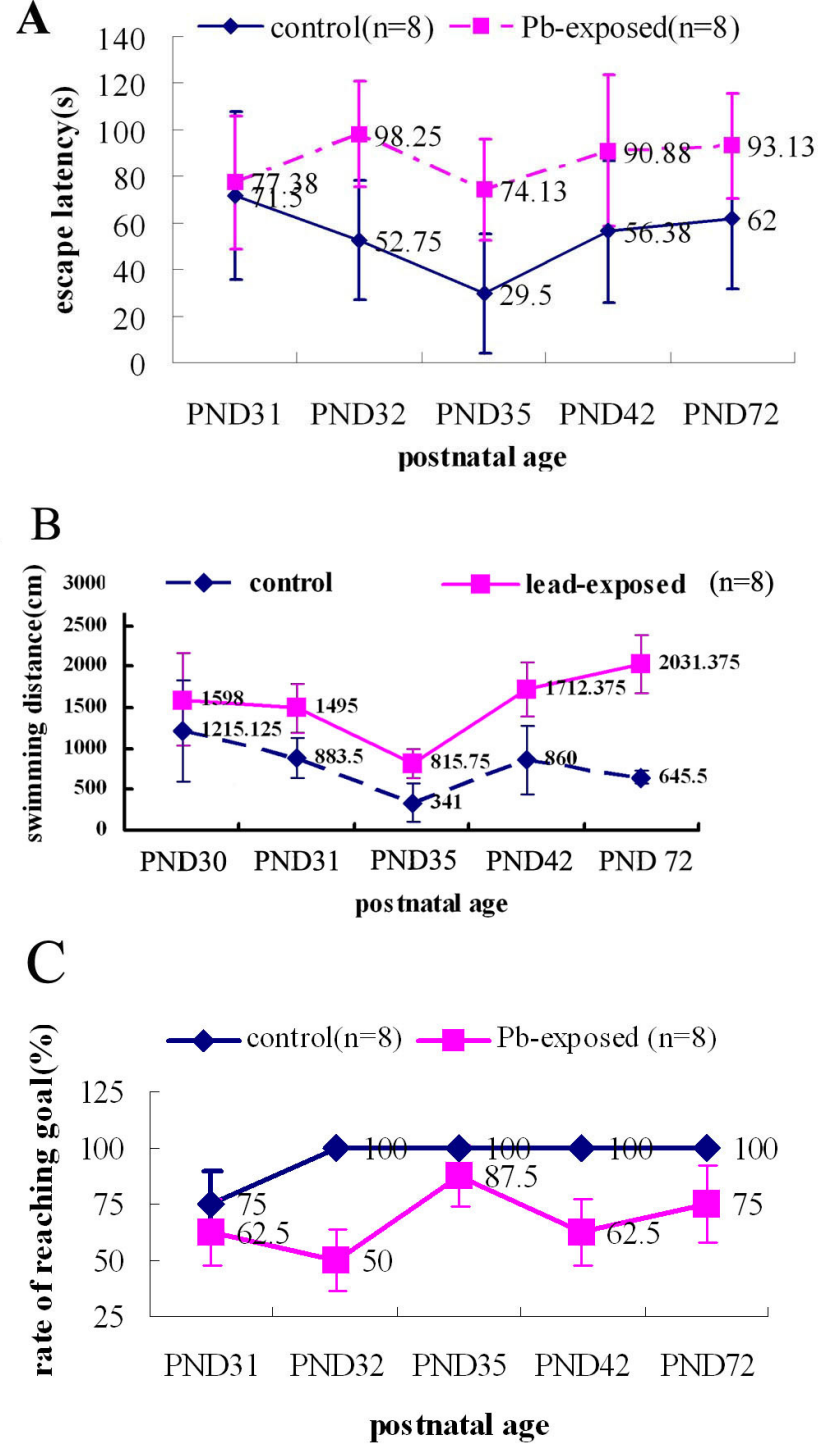
**MWM analysis of lead-exposed and control rats over 42 days of MWM acquisition revealed a statistically significant behavioral deficit**. (**A**) Escape latency (mean ± standard deviation) of the two groups. (**B**) Swimming distance (mean ± standard deviation) of the two groups. Baselines of pretraining latency and distance traveled were not significantly different between the two groups (*P *= 0.39 and *P *= 0.22, *n *= 8). With training proceeding, controls had higher learning efficiency and shorter goal latencies and distance than lead-exposed rats (*P *= 0.001 and *P *< 0.001 by repeated-measures analysis of variance, *n *= 8). (**C**) Rate (mean ± standard deviation) of reaching goal of the two groups. On PND35, PND 42, and PND 72, all the control rats found the platform within 120 seconds whereas some of lead-exposed rats failed to do so.

In testing using the MWM, the baselines of pretraining latency and swimming distance of the controls were not significantly different from that of the lead-exposed rats (respectively *F *= 0.80, *P *= 0.39 and *F *= 1.68, *P *= 0.22, *n *= 8). With training proceeding, the overall decrease in goal latency and swimming distance was taken to indicate that rats in both groups were trained to swim onto the platform, but control rats had higher learning efficiency, who had shorter goal latencies and less distance than lead-exposed rats (latency and swimming distance were respectively *P *= 0.001 and *P *< 0.001 by repeated-measures analysis of variance, *n *= 8, Figure 2A–B). On PND 35, PND 42, and PND 72, all the rats from control group found the platform within 120 seconds, whereas the lead-exposed group had a relatively lower ratio for reaching platform (see Figure 2C). More dense movement trails were observed in the target quadrant for the control group compared with the lead-exposed group.

### Expression levels of *mGluR3 *and *mGluR7 *mRNA after lead exposure

Optical-density ratios at 260 to 280 nm for total RNA were all between 1.8 and 2.0. Agarose gel electrophoresis showed that the 28S and 18S ribosomal RNA bands were clearly visible at a staining intensity of about 2:1 (28S:18S).

**Figure 3:**
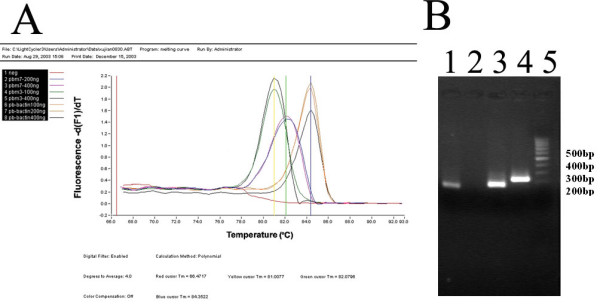
**Gene expression of *mGluR3 *and *mGluR7 *mRNA in pups' hippocampus after perinatal lead exposure**. **(A) **Melting curve analysis of SYBR green I dye PCR assay. Melting-curve analysis showed that all PCR amplifications led to a single and specific product and the melting temperatures (Tm) of all target genes were as follows: *mGluR3 *(Tm: 81.01°C), *mGluR7 *(Tm: 82.08°C), and *β-actin *(Tm: 84.35°C). **(B) **Confirmatory 2% agarose gel electrophoresis showing the target *mGluR3 *(197 bp) and *mGluR7 *(173 bp) and *β-actin *(218 bp) products. Lane 1:*mGluR7*; lane2: negative control; lane3:*mGluR3*; lane4: *β-actin*; and lane5: molecular weight markers.

By drawing standard curves for the *β-actin *gene and other targeted genes, we found a linear relationship between the cycle threshold value and the logarithm of the starting concentration of the cDNA standard. PCR efficiency of *β-actin, mGluR3 *and *mGluR7 *were respectively 1.96, 1.94 and 1.76; coefficients of variability of PCR efficiency were respectively 0, 0.2% and 0.3%; Tms were respectively 84.35°C, 81.01°C and 82.08°C, coefficients of variability of Tms were 0.21%, 0.23% and 0.37%. Melting-curve analysis showed that all PCR amplifications led to a single and specific product. Products were identified on 2% high-resolution agarose gel electrophoresis (Figure 3). Relative and normalized expression ratios for *mGluR3/β-actin *and *mGluR7/β-actin *were respectively 1.27 ± 0.26 and 0.99 ± 0.06 (a ratio of 1 indicates no change in gene expression, <1 indicates reduced expression, and >1 indicates increased expression, a ratio <0.5 or >2 is considered significant). Lead exposure of 0.2% lead acetate did not substantially change gene expression of *mGluR3 *and *mGluR7 *mRNA compared with controls.

## Discussion

Our study has assessed the impact of lead exposure during the gestational and lactational periods on gene expression of *mGluR3 *and *mGluR7 *mRNA, but significant difference of expression levels is not observed in lead-exposed rats and non-exposed controls.

In this study, lead exposure level of 0.2% lead acetate was administered to Sprague-Dawley rats as was used in most lead-exposed experiments, which was found to cause an increase in rats' blood lead levels similar to the degree of modest to severe lead poisoning in children. Thus we considered that the dose of lead exposure which has been used in this study was appropriate and the hypothesis that the exposure level of 0.2% lead acetate might be too low to reveal any obvious change in expression of *mGluR3 *and *mGluR7 *mRNA should be ruled out.

On the other hand, the putative role of G-protein-coupled metabotropic receptors in LTP and LTD has been the subject of intense investigation recently. Although recent studies demonstrated that *mGluR3 *played an essential role in LTD and a modulatory role in LTP, and functioned to regulate activity-dependent synaptic potentiation in the hippocampus [21,41], and *mGluR7 *might mediate a reduction in synaptic transmission through a mechanism such as decreasing calcium influx [19,24], the results of our studies showed that no obvious variation of *mGluR3/7 *mRNA expression occurred after pre-natal and early post-natal lead exposure. Many studies have revealed that ionotropic glutamate receptors NMDARs acted as one of targets of lead induced neurotoxicity, mainly by means of the decreased expression of NMDARs subtypes *NR2A *mRNA and *NR1 *mRNA and therefore resulting in a decrease of calcium-dependent synaptic transmission. There is still lack of studies of other factors, such as the studies of effects of lead exposure on affinity of glutamate receptors. Several scientists had done some research about the impact of lead on binding abilities of glutamate receptors and found that developmental lead exposure altered expression levels of components of NMDAR with no change in binding affinity [42,43]. The binding affinity was not considered as key elements of lead induced neurotoxicity [44,45]. In conclusion, we speculate that rat *mGluR3 *and *mGluR7 *might not involve in the pathways mediating lead neurotoxicity. A potential limitation of the present study is that the results are only from rats and lack of data of other genus yet.

In neuronal ultrastructural detection and MWM task, we found that exposure to lead before and after birth can result in ultrastructural alterations and STM deficits, which is consistent with previous results [44,45]. The hippocampus called "time window of memory" plays an especially important role in the storage of STM and the transition from STM to LTM [46-49], hippocampal ultrastructural alterations maybe one of mechanisms of lead-induced neurotoxicity. Moreover, a modified MWM procedure was applied and LTM was found also injured which was another proof that lead may cause irreversible neurological damage to neurodevelopment.

The present study suggests that lead exposure has no obvious effect on hippocampal *mGluR3 *and *mGluR7 *mRNA expression, and rat hippocampal *mGluR3 *and *mGluR7 *might not associate with lead induced neurotoxicity. Further studies are required to reveal the outcomes of another spliced variants of mGluRs after lead exposure. We believe this study is among the first to examine the role of *mGluR3 *and *mGluR7 *in lead neurotoxicity.

## Competing interests

The authors declare that they have no competing interests.

## Authors' contributions

XJ contributed to the acquisition and interpretation of the data and drafted the manuscript. YCH contributed to the design of the study, and the revision of the manuscript. YB and ZXY participated in the acquisition and the analysis of the data. TSL participated in the study conception design and interpretation of data. TY participated in the design of the study. All the authors have read and approved the final manuscript.
